# Salivary RNA Signatures in Oral Cancer Detection

**DOI:** 10.1155/2014/450629

**Published:** 2014-12-10

**Authors:** Prashanth Panta, Venkat Raghavender Venna

**Affiliations:** ^1^Department of Oral Medicine and Radiology, MNR Dental College and Hospital, Narsapur Road, Sangareddy, Telangana 502294, India; ^2^Division of Pulmonary and Critical Care Medicine, Johns Hopkins School of Medicine, 1830 East Monument Street, 5th Floor, Baltimore, MD 21205, USA

## Abstract

Oral squamous cell carcinomas (OSCC) are common malignancies that affect almost a million people every year. The key issue in reducing mortality and morbidity associated with OSCC is to develop novel strategies to identify OSCC at an early stage. One such strategy is the identification of biomarkers. So far, more than 100 biomarkers are recognized in the detection of oral cancer and they range from proteins to nucleic acids (DNAs, RNAs). Detection of ribose nucleic acids in saliva is a recent trend in diagnosing oral cancer. Studies have shown statistically significant changes in the levels of salivary transcriptomes in patients with oral squamous cell carcinomas. These biomarkers have displayed high sensitivity and specificity. Also, new point-of-care platforms such as oral fluid nanosensor test are now available that will soon emerge as chair-side tools for early detection of oral cancer. The aim of this review is to highlight the importance of salivary transcriptomes in oral cancer detection.

## 1. Introduction

**Figure 1:**
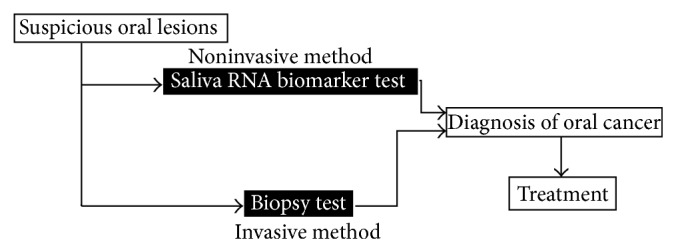
Invasive method versus noninvasive method for diagnosing oral cancer.

Saliva is a reservoir of innumerable biomolecules whose levels reflect systemic health and disease status. Therefore, saliva can be considered as a mirror of body health [1]. Saliva has been proposed as a diagnostic medium of choice because its collection is simple, noninvasive, less time consuming, and inexpensive [2]. Squamous cell carcinomas of the oral cavity are common malignancies. The low survival rates and morbidity can be attributed to the late diagnosis [3]. Hence, several new trends have been emerging that have successfully addressed this problem among which salivary RNAs are noteworthy [4]. The first report of saliva as a diagnostic medium for oral cancer was published by Liao et al. who identified mutations in exon4, condon63 of p53, in 5 out of 8 patients with oral squamous cell carcinoma [5]. Saliva can be utilized for early detection of oral cancer as this body fluid maintains continuous contact with these lesions. Diagnosis of OSCC is currently based on biopsy test, which is an invasive method. There is a need for developing a noninvasive screening tool (biomarker test) for early detection of squamous cell carcinoma (Figure 1).

In 1998, the National Institutes of Health (NIH) defined biomarker as a characteristic that is an objectively measured and evaluated indicator of normal biologic processes, pathogenic processes, or pharmacologic responses to therapeutic intervention [6]. Biomarkers are molecular signatures that are unique to a certain disease (e.g., oral cancer).

Rationale behind the use of salivary biomarkers is as follows.

1. Saliva contains a wide range of compounds.
2. Saliva is easily accessible.
3. Saliva sampling for diagnosis will improve patient comfort, as it is a noninvasive method of disease detection [2].
4. Saliva samples are safe to handle. It has biomolecules that can inhibit human immunodeficiency virus, and hence the chances of transmission are very low as compared to transmission from blood samples [6].
5. As compared to blood samples, saliva samples are easy to store, as saliva does not clot [6].

Despite several advantages of salivary biomarker estimation disadvantages do exist. The concentration of most analytes in saliva is very low (100–1000-fold) as compared to their concentration in blood [6]. However, with reference to saliva sampling in oral cancer, this may not be a limitation as the biomarkers are usually locally released from the tumor site.

The salivary biomarkers for oral cancer can be broadly divided into protein and RNA based biomarkers. Protein based biomarkers include a group of biomarkers such as cytokines, fibroblast growth factor, cyfra 21-1, cancer antigen-125, tissue polypeptide antigen, endothelin, matrix metalloproteinases, glutathione transferase, and superoxide dismutase [1]. RNA based biomarkers include a group of recently discovered biomarkers, which include messenger RNAs and microRNAs. In this review, we present the importance of ribose nucleic acids in saliva and their role as biomarkers in the diagnosis of oral squamous cell carcinoma.

## 2. Ribose Nucleic Acids in Saliva

In oral squamous cell carcinoma subjects, the transcription of specific mRNAs and miRNAs is altered (Table 1). For years, RNAs were believed to quickly degrade in the saliva due to the effect of salivary ribonucleases. However, several studies have confirmed that RNAs in saliva can exist as stable molecules [7, 8]. It is now proven that salivary RNAs are protected from degradation by cell organelles called exosomes. Exosomes are fundamentally membrane bound cell organelles whose size ranges from 30 to 100 nm in diameter. They originate from the endoplasmic reticulum and are abundantly filled with mRNAs, miRNAs [9–11]. Skog et al. and Al-Nedawi et al. have concluded that their exocytosis is one mechanism for accumulation of ribose nucleic acids in saliva [12, 13]. So, their careful profiling provides insight into the diagnosis of oral cancer. The ribose nucleic acids (RNAs) in the saliva are produced either locally or from serum [14–16]. Serum derived RNAs are transported via acinar cells and gingival crevicular fluid and also by transcellular (active transport or passive diffusion) and paracellular routes (ultrafiltration) [17]. Cellular necrosis and apoptosis are believed to be the principal mechanisms of release of ribose nucleic acids in saliva [18, 19].

## 3. Messenger RNA Profile in Oral Cancer

Messenger RNAs are a family of molecules that convey information from the DNA to the ribosome. This information is in the form of a sequence of nucleotides arranged into codons that denote specific amino acids. With the help of transfer RNA and ribosomal RNA, translation occurs at the level of the ribosome, wherein the mRNA molecule is converted into a polymer of amino acids. Several researches have shown that the levels of mRNA reflect physiological states and disease processes. Li and his associates were the first group of investigators, who have shown that saliva supernatant from healthy individuals contains more than 3000 mRNA species [20]. Li et al. have found in a series of experiments that various mRNAs are upregulated in the saliva of patients suffering from oral squamous cell carcinoma (OSSC) [5, 20]. A statistical significance elevation was noted in seven mRNA transcripts that include DUSP1, H3F3A, IL IB, IL8, OAZ1, SAT, and S100 P. This study was conducted using microarray analysis and quantitative PCR in a sample size of 64 individuals consisting of 32 controls and 32 patients with OSCC. These biomarkers displayed sensitivity and specificity of 91% when used in combination [5]. Although 1670 genes exhibited differential expression, among them only 7 transcripts demonstrated statistical significance. Recently, studies were conducted on much larger sample sizes (150 patients) by Wong and his associates, further validating these 7 mRNA transcriptomes.

Studies conducted by most of the investigators identified a significant elevation in 7 mRNA molecules. They include the following.

*DUSP1.* Dual specificity protein phosphatase 1 is an enzyme that is encoded by the DUSP1 gene situated on chromosome 5 [21, 22]. This gene spans about 3111 bp and is made up of four exons separated by three introns. Transcription of this gene results in an mRNA whose expression plays an important role in activating MAPK pathway that participates in protein modification, oxidative stress, and signal transduction [23, 24]. DUSP1 is further controlled by the p53 gene and hypermethylation of DUSP1 gene is a necessary event in oral carcinogenesis [25].

*H3F3A.* H3 Histone, Family 3A, is a protein that is encoded by the H3F3A gene situated on chromosome 1 [26]. Histones in general are nuclear proteins responsible for the structural integrity of chromosomal nucleosome. H3F3A gene is made up of 5 exons spanning about 9282 bp. The H3F3A mRNA is a proliferative marker made up of 135 amino acids and weighs 15 kd [5].

*IL IB.* Interleukin 1 Beta is a member of Interleukin 1 family of cytokines [27]. This protein is an important mediator of inflammation, cell proliferation, differentiation, and apoptosis. The gene that corresponds to this protein is situated on chromosome 2. Studies have shown elevated serum levels of IL IB in patients with oral squamous cell carcinoma [28].

*IL8.* Interleukin 8 is a proinflammatory cytokine also known as neutrophil chemotactic factor [29]. This gene is located on chromosome 4 and spans 3211 bp. After translation, an mRNA is produced that contains 99 amino acids and weighs 11 Kd. It plays a key role in tumor angiogenesis, cell adhesion, immunity, and cell cycle arrest [30]. St. John et al have assessed the expression of IL-8 in serum and saliva at the messenger RNA and protein levels. They have concluded that IL 8 in saliva (*P* < 0.01) holds promise as a biomarker for OSCC [31]. Electrochemical sensors were used for detection and ROC analysis for predictive power estimation. This test has yielded high sensitivity and specificity for both IL8 and IL8 mRNA alone and in combination.

*OAZ1.* The ornithine decarboxylase antizyme 1 is a protein that plays an important role in the regulation of polyamine synthesis [32]. The OAZ1 gene is located on chromosome 19 [33] and spans 3969 bp. Its gene product contains 228 amino acids and inhibits ornithine decarboxylase and is thought to be a tumor suppressor [5].

*SAT.* Spermidine/Spermine N1-Acetyltransferase 1 is a protein encoded by the gene SAT [34]. This protein belongs to the acetyl transferase family and participates in the catabolism of polyamines [35]. This gene is situated on chromosome X and is made up of 4095 bp.

*S100 P.* S100 calcium binding protein P is a member of the S100 family of proteins that is made up of 95 amino acids [36]. The S100 proteins located either in the cytoplasm or in the nucleus that participate in cell cycle regulation and differentiation. The gene that encodes this protein is situated on chromosome 4.

## 4. MicroRNA Profile in Oral Cancer

MicroRNAs (miRNAs) are short RNA transcripts that range from 19 to 25 nucleotides [37, 38]. They were discovered during the early 90s in a transparent nematode. Until now more than 1000 miRNAs have been profiled and their dysregulation was found to affect cell growth, apoptosis, differentiation, motility, and also immunity [39, 40]. With relevance to oral cancer miRNAs have shown to play a significant role in cell proliferation and apoptosis [41, 42]. MicroRNAs can be potential biomarkers in the diagnosis of oral cancer as their expression increases to a significant proportion even several times (10–100-fold increase) as compared to mRNAs. Studies conducted by Park and his associates have found significantly reduced levels of miR-125a and miR-200a in the saliva of oral cancer patients as compared to healthy controls [43]. The expressions of 50 miRNAs were studied, but only 2 miRNAs demonstrated downregulation. In another study, Liu et al. have explored the clinical application of miR-31 as a biomarker in oral cancer [44]. It was shown in their study that levels of miR-31 were higher in saliva as compared to blood, indicating that they are released locally from the tumor site. Studies conducted by most of the investigators identified a significant change in 3 miRNA molecules. They include the following.

*miR-125a.* The gene that encodes the miR-125a is made up of 86 bases and is situated on chromosome 19. miR-125a plays an important role in cell proliferation and can effect the genes involved in MAPK metabolism [45].

*miR-200a.* The gene that encodes microRNA-200a is situated on chromosome 1 and there is sufficient evidence that it is involved in tumor suppression and early metastasis [46]. The miR-200a belongs to the miR-200 family of short RNA molecules that include miR-200a, miR-200b, miR-200c, miR-141, and miR-429 arranged in two clusters. Its levels are downregulated during metastasis and can be inversely correlated to the degree of invasion. Aslakson and Miller have conducted a study on cell lines that displayed metastatic potential [47]. These cell lines were injected into the mammary fat pad of mice and their metastatic ability was studied. In their study, a clear distinction could be made between nonmetastatic and highly metastatic cell lines based on the expression of miR-200.

*miR-31.* miR-31 is coded by the gene made up of 71 bases and is situated on chromosome 9. This is a tumor suppressor microRNA, whose levels are completely lost in metastatic oral tumors. Genetic experiments conducted by Liu et al. have shown that salivary miR-31 was significantly elevated in all the stages of oral cancer irrespective of the tumor size [44]. Lajer et al. used 51 biopsies and subjected them to microarray analysis [48]. They found that the most significant perturbation between the carcinoma group and the control group was the upregulation of miR-31.

## 5. Principle Methods Employed in the Detection of Salivary Ribose Nucleic Acids

During the early stages of research, RNA isolation was a costly affair but recently several simple methods have been developed. Quantitative polymerase chain reaction (qPCR) and microarrays (proven gold standard) followed by qPCR are the principle methods used in salivary RNA analysis.

### 5.1. Microarray Technology

**Figure 2:**
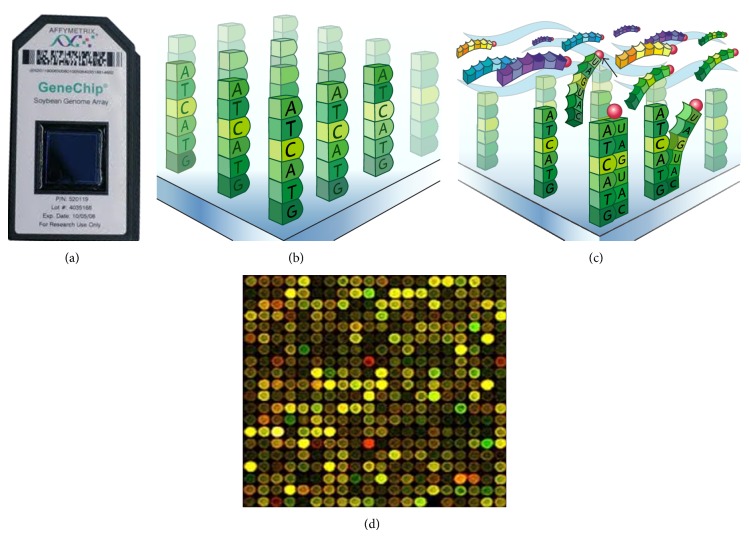
(a) Microarray chip. (b) Oligonucleotides on a solid base. (c) Hybridization between fluorescently labeled target sequences and probe sequences. (d) A digital image.

Microarray technology allows researchers to investigate the expression profile of a large set of genes. Microarray is a collection of several miniature spots of specific DNA sequences (oligonucleotides) located on a solid base (glass, silicon chip, and microscopic beads) (Figures 2(a) and 2(b)) [49]. Each of such sequences is known as a probe and allows hybridization of cDNA or cRNA from a sample. Hybridization between two strands of nucleic acids via hydrogen bonds is the working principle behind microarray analysis (Figure 2(c)). The sequences (cDNA or RNA) in the sample under study are firstly fused to fluorescent dyes (cyanine 3, cyanine 5) [50]. The fluorescently labeled target sequences (Figure 2(c)) bind to the probe sequences (hybridization) and generate a signal whose strength is measured. The measurement is in terms of the number of photons emitted after stimulation with a laser of a particular wavelength. A digital image (Figure 2(d)) is formed and later intensity values are obtained for each probe set. Microarray manufacturers provide data analysis software (e.g., microarray suite software) along with plate readers that help in the creation of raw data. Later, this is subjected to background correction, quality control measures, spot filtration, aggregation, and normalization followed by identification of gene expression and pattern recognition. Since the inception of RNA transcriptome analysis, a variety of new microarray platforms have evolved of which “Affymetrix U133 Plus 2.0” and “Human Exon 1.0 ST (HuEx)” have been of great value in RNA research. “Affymetrix U133 Plus 2.0” is a quartz gene chip that can be used to detect specific gene expression by detecting RNA transcripts. It contains 54000 probe sets and 1,300,000 distinct oligonucleotide features representing approximately 38500 genes. The HuEx is another novel microarray platform capable of making high throughput measurements of gene expression of nearly 28869 human genes.

### 5.2. Quantitative Polymerase Chain Reaction

Quantitative polymerase chain reaction (qPCR) in RNA analysis is a technique with which the amount of an expressed gene can be measured (underexpressed or overexpressed) based on the amount of mRNA in the sample. Before this step, the RNA sample should be reverse transcribed to complementary DNA (template). This method is a modification of polymerase chain reaction and is carried out in a thermal cycler. The sample is mixed with sequence specific probes that are intercalated to fluorescent molecules known as “reporters.” The fluorophore labeled sequence gets hybridized to the complementary sequence. The thermal cycler has the capacity to illuminate each sample with a light of specific wavelength and the sensors in it detect the fluorescence emitted by the excited fluorophore. The instrument heats and chills the samples in cycles that can roughly be divided into three stages. In the first stage, the nucleic acid double chain is separated; in the second one there will be binding of primers; and in the third one, polymerization of DNA takes place. Quantification of gene expression is primarily by two methods: relative quantification and absolute quantification [51]. Absolute quantification gives the exact number of DNA molecules and relative quantification determines fold differences in the expression of a specific gene. This method can be used to quantify the amount of mRNA and miRNA in an unknown saliva sample.

### 5.3. Nanotechnology/Point-of-Care Platforms

Oral fluid nanosensor test (OFNASET) is a very recent point-of-care technology that was developed by Dr. Wong. This “lab on chip” is an automated, easy to use, cost-effective device that can detect about 8 biomarkers in 15 minutes [52]. This system works on the principle of electrochemical detection of salivary proteomes and transcriptomes. Screening can be made in patients with a risk of oral cancer, who can later be referred for biopsies.

## 6. Conclusion

Saliva collection unlike blood and tissue sampling is a noninvasive and a reliable method for diagnosing oral cancer. Currently, a biopsy test (gold standard) is mandatory for diagnosing oral cancer. But, in the near future, salivary biomarkers may replace biopsy. It is now proven that the levels of dual specificity protein phosphatase 1, H3 Histone, Family 3A, Interleukin 1 Beta, Interleukin 8, ornithine decarboxylase antizyme 1, Spermidine N1-Acetyltransferase 1, S100 calcium binding protein P, and miR-31 are upregulated and the levels of miR-125a and miR-200a are downregulated in oral cancer patients. Several new point-of-care technologies have been emerging in salivary RNA analysis and monitoring RNAs in saliva may soon become a screening test and a diagnostic tool for oral cancer.

## Figures and Tables

**Table 1:** Various salivary transcriptomes and their role in oral carcinogenesis.

Transcriptome	Role in metabolism and oral carcinogenesis
DUSP1	DUSP mRNA participates in the MAPK pathway. Hypermethylation of DUSP1 gene is associated with oral carcinogenesis [23–25].
H3F3A	It maintains the integrity of chromosomal nucleosome and is a proliferative marker [5, 26].
IL IB	It is chemical mediator of cell proliferation, differentiation, and apoptosis [27, 28].
IL 8	It is a player in tumor angiogenesis, cell adhesion, and cell cycle arrest [29–31].
OAZ1	It regulates polyamine synthesis and is a tumor suppressor [5, 32, 33].
SAT	It is responsible for catabolism of polyamines [35].
S100 P	It is responsible for cell cycle regulation and differentiation [36].
miR-125a	It affects genes in MAPK pathway and in cell proliferation [45].
miR-200a	It plays an important role in tumor suppression and early metastasis [46, 47].
miR-31	This is a tumor suppressor [44, 48].

## References

[1] Shah F. D., Begum R., Vajaria B. N., Patel K. R., Patel J. B., Shukla S. N., Patel P. S. (2011). A review on salivary genomics and proteomics biomarkers in oral cancer. *Indian Journal of Clinical Biochemistry*.

[2] Denny P., Hagen F. K., Hardt M., Liao L., Yan W., Arellanno M., Bassilian S., Bedi G. S., Boontheung P., Cociorva D., Delahunty C. M., Denny I., Dunsmore J., Faull K. F., Gilligan J., Gonzalez-Begne M., Halgand F., Hall S. C., Han X., Henson B., Hewel J., Hu S., Jeflrey S., Jiang J., Loo J. A., Ogorzalek Loo R. R., Malamud D., Melvin J. E., Miroshnychenko O., Navazesh M., Niles R., Park S. K., Prakobphol A., Ramachandran P., Richert M., Robinson S., Sondej M., Souda P., Sullivan M. A., Takashima J., Than S., Wang J., Whitelegge J. P., Witkowska H. E., Wolinsky L., Xie Y., Xu T., Yu W., Ytterberg J., Wong D. T., Yates J. R., Fisher S. J. (2008). The proteomes of human parotid and submandibular/sublingual gland salivas collected as the ductal secretions. *Journal of Proteome Research*.

[3] Peacock Z. S., Pogrel M. A., Schmidt B. L. (2008). Exploring the reasons for delay in treatment of oral cancer. *Journal of the American Dental Association*.

[4] Li Y., St. John M. A. R., Zhou X., Kim Y., Sinha U., Jordan R. C. K., Eisele D., Abemayor E., Elashoff D., Park N.-H., Wong D. T. (2004). Salivary transcriptome diagnostics for oral cancer detection. *Clinical Cancer Research*.

[5] Liao P.-H., Chang Y.-C., Huang M.-F., Tai K.-W., Chou M.-Y. (2000). Mutation of *p53* gene codon 63 in saliva as a molecular marker for oral squamous cell carcinomas. *Oral Oncology*.

[6] Yoshizawa J. M., Schafer C. A., Schafer J. J., Farrell J. J., Paster B. J., Wong D. T. W. (2013). Salivary biomarkers: toward future clinical and diagnostic utilities. *Clinical Microbiology Reviews*.

[7] Tsui N. B. Y., Ng E. K. O., Lo Y. M. D. (2002). Stability of endogenous and added RNA in blood specimens, serum, and plasma. *Clinical Chemistry*.

[8] El-Hefnawy T., Raja S., Kelly L. (2004). Characterization of amplifiable, circulating RNA in plasma and its potential as a tool for cancer diagnostics. *Clinical Chemistry*.

[9] Théry C. (2011). Exosomes: secreted vesicles and intercellular communications. *F1000 Biology Reports*.

[10] García J. M., García V., Peña C., Domínguez G., Silva J., Diaz R., Espinosa P., Citores M. J., Collado M., Bonilla F. (2008). Extracellular plasma RNA from colon cancer patients is confined in a vesicle-like structure and is mRNA-enriched. *RNA*.

[11] Yuan A., Farber E. L., Rapoport A. L. (2009). Transfer of microRNAs by embryonic stem cell microvesicles. *PLoS ONE*.

[12] Skog J., Würdinger T., van Rijn S., Meijer D. H., Gainche L., Curry W. T., Carter B. S., Krichevsky A. M., Breakefield X. O. (2008). Glioblastoma microvesicles transport RNA and proteins that promote tumour growth and provide diagnostic biomarkers. *Nature Cell Biology*.

[13] Al-Nedawi K., Meehan B., Rak J. (2009). Microvesicles: messengers and mediators of tumor progression. *Cell Cycle*.

[14] Haeckel R., Hanecke P. (1996). Application of saliva for drug monitoring: an in vivo model for transmembrane transport. *European Journal of Clinical Chemistry and Clinical Biochemistry*.

[15] Lawrence H. P. (2002). Salivary markers of systemic disease: noninvasive diagnosis of disease and monitoring of general health. *Journal of the Canadian Dental Association*.

[16] Kaufman E., Lamster I. B. (2002). The diagnostic applications of saliva—a review. *Critical Reviews in Oral Biology and Medicine*.

[17] Baum B. J. (1993). Principles of saliva secretion. *Annals of the New York Academy of Sciences*.

[18] Ratajczak J., Wysoczynski M., Hayek F., Janowska-Wieczorek A., Ratajczak M. Z. (2006). Membrane-derived microvesicles: important and underappreciated mediators of cell-to-cell communication. *Leukemia*.

[19] Aps J. K. M., Martens L. C. (2005). Review: the physiology of saliva and transfer of drugs into saliva. *Forensic Science International*.

[20] Li Y., Zhou X., St. John M. A. R., Wong D. T. W. (2004). RNA profiling of cell-free saliva using microarray technology. *Journal of Dental Research*.

[21] Keyse S. M., Emslie E. A. (1992). Oxidative stress and heat shock induce a human gene encoding a protein-tyrosine phosphatase. *Nature*.

[22] Martell K. J., Kwak S., Hakes D. J., Dixon J. E., Trent J. M. (1994). Chromosomal localization of four human VH1-like protein-tyrosine phosphatases. *Genomics*.

[23] Tanoue T., Yamamoto T., Maeda R., Nishida E. (2001). A novel MAPK phosphatase MKP-7 acts preferentially on JNK/SAPK and p38*α* and *β* MAPKs. *The Journal of Biological Chemistry*.

[24] Slack D. N., Seternes O.-M., Gabrielsen M., Keyse S. M. (2001). Distinct binding determinants for ERK2/p38*α* and JNK map kinases mediate catalytic activation and substrate selectivity of map kinase phosphatase-1. *The Journal of Biological Chemistry*.

[25] Khor G. H., Froemming G. R. A., Zain R. B. (2013). DNA methylation profiling revealed promoter hypermethylation-induced silencing of p16, DDAH2 and DUSP1 in primary oral squamous cell carcinoma. *International Journal of Medical Sciences*.

[26] Zhang Y., Reinberg D. (2001). Transcription regulation by histone methylation: Interplay between different covalent modifications of the core histone tails. *Genes and Development*.

[27] Bensi G., Raugei G., Palla E., Carinci V., Buonamassa D. T., Melli M. (1987). Human interleukin-1 beta gene. *Gene*.

[28] Arellano-Garcia M. E., Hu S., Wang J. (2008). Multiplexed immunobead-based assay for detection of oral cancer protein biomarkers in saliva. *Oral Diseases*.

[29] Modi W. S., Dean M., Seuanez H. N., Mukaida N., Matsushima K., O'Brien S. J. (1990). Monocyte-derived neutrophil chemotactic factor (MDNCF/IL-8) resides in a gene cluster along with several other members of the platelet factor 4 gene superfamily. *Human Genetics*.

[30] van Damme J., Rampart M., Conings R. (1990). The neutrophil-activating proteins interleukin 8 and *β*-thromboglobulin: *in vitro* and *in vivo* comparison of NH_2_-terminally processed forms. *European Journal of Immunology*.

[31] St. John M. A. R., Li Y., Zhou X., Denny P., Ho C.-M., Montemagno C., Shi W., Qi F., Wu B., Sinha U., Jordan R., Wolinsky L., Park N.-H., Liu H., Abemayor E., Wong D. T. W. (2004). Interleukin 6 and interleukin 8 as potential biomarkers for oral cavity and oropharyngeal squamous cell carcinoma. *Archives of Otolaryngology—Head and Neck Surgery*.

[32] Tewari D. S., Qian Y., Thornton R. D., Pieringer J., Taub R., Mochan E., Tewari M. (1994). Molecular cloning and sequencing of a human cDNA encoding ornithine decarboxylase antizyme. *Biochimica et Biophysica Acta: Protein Structure and Molecular Enzymology*.

[33] Matsufuji S., Inazawa J., Hayashi T., Miyazaki Y., Ichiba T., Furusaka A., Matsufuji T., Atkins J. F., Gesteland R. F., Murakami Y., Hayashi S.-I. (1996). Assignment of the human antizyme gene (OAZ) to chromosome 19p13.3 by fluorescence in situ hybridization. *Genomics*.

[34] Xiao L., Paul C., Mank A. R., Griffin C., Jabs E. W., Hawkins A. L., Casero R. A. (1992). Structure of the human spermidine/spermine N^1^-acetyltransferase gene. Kxon/intron gene organization and localization to Xp22.1. *Biochemical and Biophysical Research Communications*.

[35] Coleman C. S., Pegg A. E. (2001). Polyamine analogues inhibit the ubiquitination of spermidine/spermine $N^{1}$-acetyltransferase and prevent its targeting to the proteasome for degradation. *Biochemical Journal*.

[36] Schäfer B. W., Heizmann C. W. (1996). The S100 family of EF-hand calcium-binding proteins: functions and pathology. *Trends in Biochemical Sciences*.

[37] Bartel D. P. (2009). MicroRNAs: target recognition and regulatory functions. *Cell*.

[38] Bartel D. P. (2004). MicroRNAs: genomics, biogenesis, mechanism, and function. *Cell*.

[39] Zeng Y. (2006). Principles of micro-RNA production and maturation. *Oncogene*.

[40] Lu J., Getz G., Miska E. A., Alvarez-Saavedra E., Lamb J., Peck D., Sweet-Cordero A., Ebert B. L., Mak R. H., Ferrando A. A., Downing J. R., Jacks T., Horvitz H. R., Golub T. R. (2005). MicroRNA expression profiles classify human cancers. *Nature*.

[41] Sekuklu S. D., Donoghue M. T. A., Spillane C. (2009). miR-21 as a key regulator of oncogenic processes. *Biochemical Society Transactions*.

[42] Wong T.-S., Liu X.-B., Wong B. Y.-H., Ng R. W.-M., Yuen A. P.-W., Wei W. I. (2008). Mature miR-184 as potential oncogenic microRNA of squamous cell carcinoma of tongue. *Clinical Cancer Research*.

[43] Park N. J., Zhou H., Elashoff D. (2009). Salivary microRNA: discovery, characterization, and clinical utility for oral cancer detection. *Clinical Cancer Research*.

[44] Liu C.-J., Lin S.-C., Yang C.-C., Cheng H.-W., Chang K.-W. (2012). Exploiting salivary miR-31 as a clinical biomarker of oral squamous cell carcinoma. *Head and Neck*.

[45] Herrera B. M., Lockstone H. E., Taylor J. M., Wills Q. F., Kaisaki P. J., Barrett A., Camps C., Fernandez C., Ragoussis J., Gauguier D., McCarthy M. I., Lindgren C. M. (2009). MicroRNA-125a is over-expressed in insulin target tissues in a spontaneous rat model of Type 2 Diabetes. *BMC Medical Genomics*.

[46] Korpal M., Lee E. S., Hu G., Kang Y. (2008). The miR-200 family inhibits epithelial-mesenchymal transition and cancer cell migration by direct targeting of E-cadherin transcriptional repressors ZEB1 and ZEB2. *The Journal of Biological Chemistry*.

[47] Aslakson C. J., Miller F. R. (1992). Selective events in the metastatic process defined by analysis of the sequential dissemination of subpopulations of a mouse mammary tumor. *Cancer Research*.

[48] Lajer C. B., Nielsen F. C., Friis-Hansen L. (2011). Different miRNA signatures of oral and pharyngeal squamous cell carcinomas: a prospective translational study. *British Journal of Cancer*.

[49] Maskos U., Southern E. M. (1992). Oligonucleotide hybridisations on glass supports: a novel linker for oligonucleotide synthesis and hybridisation properties of oligonucleotides synthesised *in situ*. *Nucleic Acids Research*.

[50] Shalon D., Smith S. J., Brown P. O. (1996). A DNA microarray system for analyzing complex DNA samples using two-color fluorescent probe hybridization. *Genome Research*.

[51] Dhanasekaran S., Doherty T. M., Kenneth J. (2010). Comparison of different standards for real-time PCR-based absolute quantification. *Journal of Immunological Methods*.

[52] Wei F., Wong D. T. W. (2012). Point-of-care platforms for salivary diagnostics. *The Chinese Journal of Dental Research*.

